# Identification of NINJ1 as a novel prognostic predictor for retroperitoneal liposarcoma

**DOI:** 10.1007/s12672-024-01016-x

**Published:** 2024-05-11

**Authors:** Yu Zhao, Da Qin, Xiangji Li, Tiange Wang, Tong Zhang, Xiaosong Rao, Li Min, Zhiyi Wan, Chenghua Luo, Mengmeng Xiao

**Affiliations:** 1 Department of Gastroenterology, Beijing Friendship Hospital, Capital Medical University, National Clinical Research Center for Digestive Disease, Beijing Digestive Disease Center, Beijing Key Laboratory for Precancerous Lesion of Digestive Disease, Beijing, China; 2 Department of Retroperitoneal Tumor Surgery, International Hospital, Peking University, Beijing, China; 3 Department of Pathology, International Hospital, Peking University, Beijing, China; 4 College of Biological Sciences, China Agricultural University, Beijing, China; 5 Department of Retroperitoneal Tumor Surgery, Peking University People’s Hospital, Beijing, China

**Keywords:** NINJ1, Prognosis, Retroperitoneal liposarcoma, Gene expression, Overall survival

## Abstract

**Background:**

Retroperitoneal liposarcoma (RPLS) is known for its propensity for local recurrence and short survival time. We aimed to identify a credible and specific prognostic biomarker for RPLS.

**Methods:**

Cases from The Cancer Genome Atlas (TCGA) sarcoma dataset were included as the training group. Co-expression modules were constructed using weighted gene co-expression network analysis (WGCNA) to explore associations between modules and survival. Survival analysis of hub genes was performed using the Kaplan–Meier method. In addition, independent external validation was performed on a cohort of 135 Chinese RPLS patients from the REtroperitoneal SArcoma Registry (RESAR) study (NCT03838718).

**Results:**

A total of 19 co-expression modules were constructed based on the expression levels of 26,497 RNAs in the TCGA cohort. Among these modules, the green module exhibited a positive correlation with overall survival (OS, *p* = 0.10) and disease-free survival (DFS, *p* = 0.06). Gene set enrichment analysis showed that the green module was associated with endocytosis and soft-tissue sarcomas. Survival analysis demonstrated that *NINJ1*, a hub gene within the green module, was positively associated with OS (*p* = 0.019) in the TCGA cohort. Moreover, in the validation cohort, patients with higher NINJ1 expression levels displayed a higher probability of survival for both OS (*p* = 0.023) and DFS (*p* = 0.012). Multivariable Cox analysis further confirmed the independent prognostic significance of NINJ1.

**Conclusions:**

We here provide a foundation for the establishment of a consensus prognostic biomarker for RPLS, which should not only facilitate medical treatment but also guide the development of novel targeted drugs.

**Supplementary Information:**

The online version contains supplementary material available at 10.1007/s12672-024-01016-x.

## Introduction

Soft-tissue sarcomas are a heterogeneous group of malignant mesenchymal tumors mostly arising from the embryonic mesoderm, among which liposarcoma is the most common pathological type in adults [1]. Liposarcomas are mainly located on the extremities (60%) and retroperitoneally (40%) [2]. Significant differences exist between retroperitoneal liposarcoma (RPLS) and liposarcoma of the extremities in both histological origin and biological behavior [2, 3]. However, most biomedical investigations have focused on tumors of the extremities, even though the treatment of RPLS is more problematic due to their complex anatomy, organ invasiveness, and high bleeding risk [4, 5]. Compared to liposarcomas of the extremities, RPLS is more prone to local recurrences (19%–44%) and shorter survival times [6]. Thus, there is an urgent need for a comprehensive understanding of the molecular mechanisms underlying RPLS and the identification of novel biomarkers specific to RPLS.

Previous reports provide a very limited understanding of the molecular basis of RPLS. Some well-known oncogenes, such as *MDM2* [7], *FGF-21* [8], *HMGA1* [9], *Calreticulin* [10], *NNAT* [11], *CCDC180* [12], and many miRNAs [13–15] are established as crucial for liposarcoma growth and closely correlate with an unfavorable prognosis. In RPLS, Siglec-15 and Tsp2 were also reported as showing a correlation with tumor progression and poor disease-free survival [16, 17]. However, because most studies are based on tumors of the extremities, few druggable targets or widely accepted molecular prognostic factors are available for treating RPLS. Additionally, most liposarcoma biomarker studies have been based on a subjective and arbitrary selection of candidate genes without a systematic biomarker screening process, which has resulted in most of the proposed biomarkers being classical epithelial cancer biomarkers [18, 19]. Meanwhile, most systematic biomarker screens have been performed on mixed sarcoma cohorts of various pathological types. Considering the unique histological origin of RPLS, biomarkers specific to RPLS have potentially been overlooked. Here, we aimed to identify a credible and specific prognostic biomarker for RPLS.

## Methods

### Patients and data

**Fig. 1:**
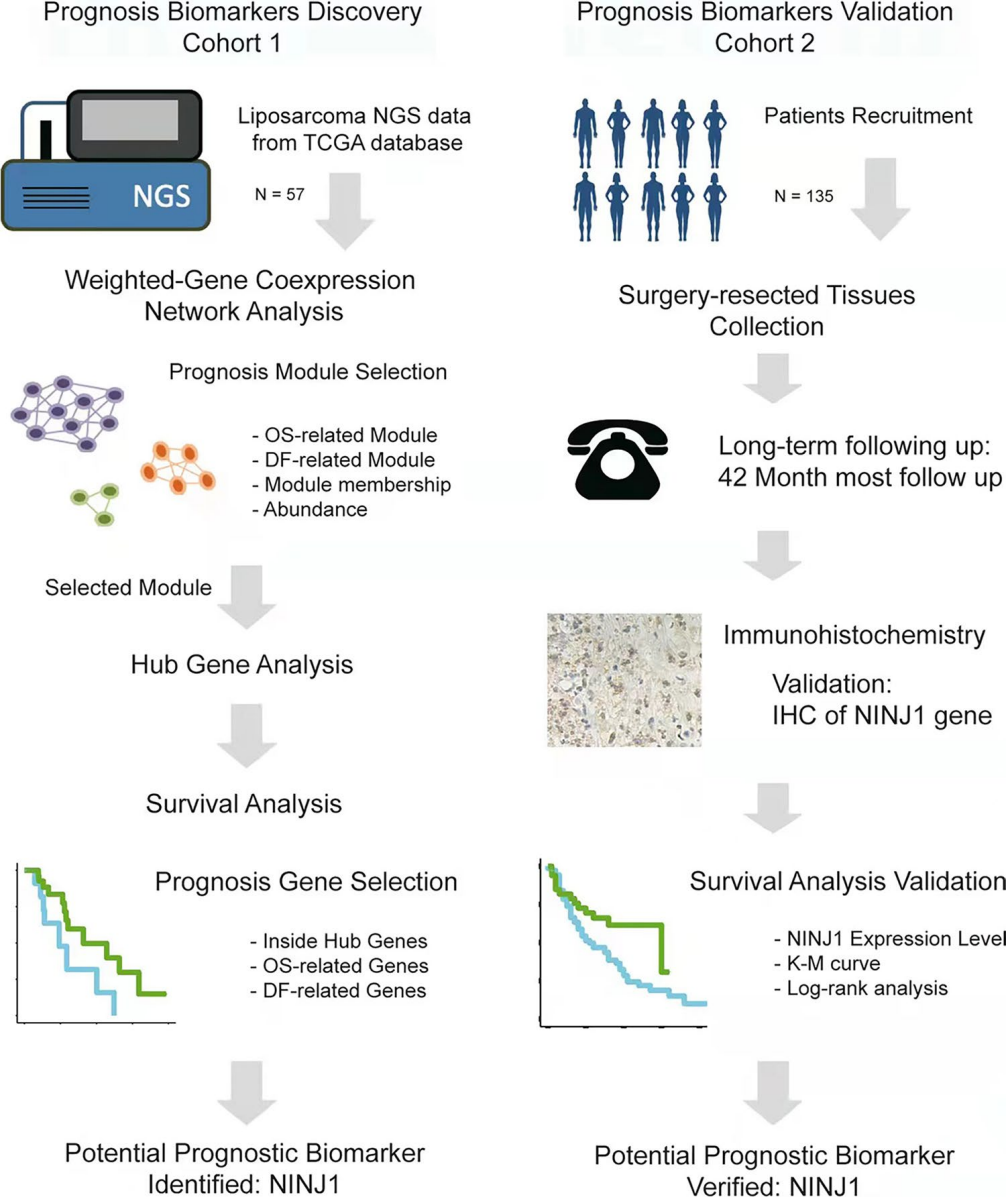
Study flowchart

In this retrospective cohort study, our training group consisted of 57 cases from the liposarcoma sub-cohort of The Cancer Genome Atlas (TCGA) database [20], which is mostly based on Caucasians. Our external verification group consisted of 135 Asian cases from the global multicenter REtroperitoneal SArcoma Registry (RESAR) study (NCT03838718), which was conducted in the Department of Retroperitoneal Tumor Surgery, Peking University International Hospital and approved by Peking University International Hospital Institutional Review Board. This study was conducted according to the guidelines of the Declaration of Helsinki and approved by the ethics committee of the International Hospital, Peking University. All participants provided written informed consent to participate in this study. The study flowchart is shown in Fig. 1. We designed a step-wise screening pipeline to identify proteins not only closely related to prognosis but also concerning biological essentiality.

### Weighted gene co-expression network analysis (WGCNA)

WGCNA was performed on the training group (TCGA cohort) using the WGCNA package in R. Genes with expression patterns determined to be similar by WGCNA were classified into a specific module, shown here with a unique color. Principal components analysis (PCA) was next applied to find the first principal component of each module. In each module, we used the first principal component from PCA, here named the module eigengene (ME), to represent the overall expression level. Subsequently, correlation analysis was conducted between the ME and various patient survival parameters, such as overall survival (OS) and disease-free survival (DFS).

Gene ontology (GO) analysis was then performed to identify ontologies (MF, molecular functions; BP, biological processes; CC, cellular component) enriched in genes in the selected module using the “clusterProfiler” package. Gene set enrichment analysis (GSEA) was then applied to identify potential pathways affected by genes in the selected module, again using the “clusterProfiler” package. Hub genes in the module were selected using the betweenness calculation of the Cytoscape software.

### Survival analysis

Kaplan–Meier (KM) analysis was then performed to evaluate the prognostic roles of hub genes using the KM “survival” and “ggplot2” packages. *P* values < 0.05 were significant.

### Immunohistochemistry (IHC)

NINJ1 antibodies were purchased from BIOSS (bs-11105R). IHC data were available, and all samples were divided into three groups based on the staining scores. The scoring system for staining extent was as follows: 1 for 0–33%, 2 for 34–66%, and 3 for 67–100%. The staining intensity was evaluated as low expression, medium expression, and high expression, which are denoted 1, 2, and 3, respectively.

## Results

### Biomarker identification

**Fig. 2:**
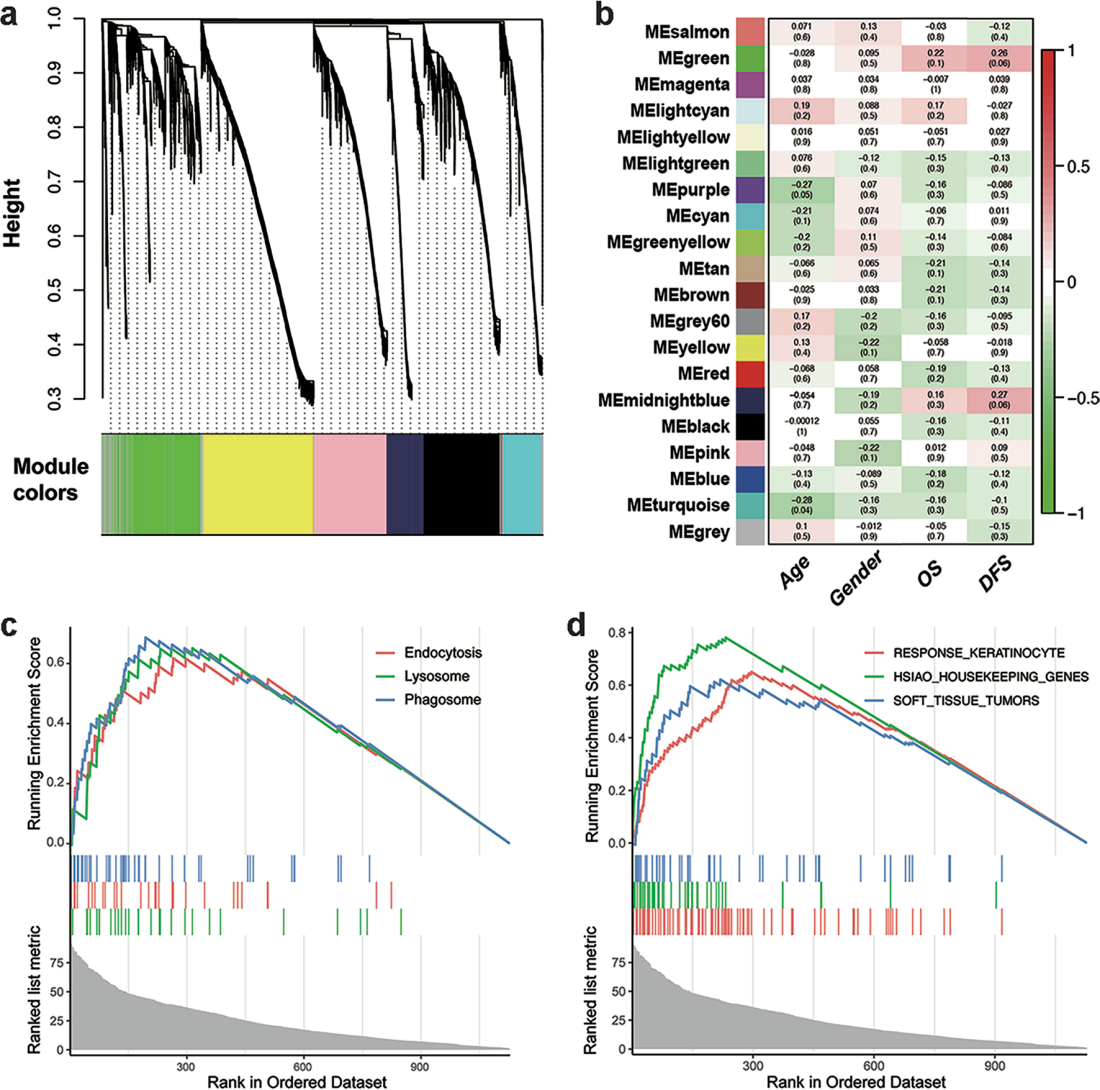
Weighted gene co-expression network analysis of liposarcoma gene-expression data from the TCGA database and discovery of prognosis-related modules in the training cohort. **a** Genes with similar expression patterns are clustered into a unique module. The clustering pattern in several modules is shown. **b** Heatmap of the relationship between a module and clinical features. Red, a positive correlation; Green, a negative correlation. Numbers in the heatmap represent the correlation coefficient (p-value). OS, overall survival; DFS, disease-free survival. Gene set enrichment analysis shows that the green module is related to endocytosis (**c**) and soft-tissue tumors (**d**)

In the TCGA cohort, a total of 19 co-expression modules were constructed from the gene expression matrix, which was based on the expression levels of 26,497 RNAs (Fig. 2a). Detailed parameters and sample clustering by WGCNA are shown in Fig. S1a, b. Among these modules, turquoise, blue, brown, and yellow were four major modules with more than 1000 genes (Supplementary Fig. 1c). Notably, we observed a positive correlation between the green module (MEgreen) and prognosis, including OS (Fig. 2b, r = 0.22, *p* = 0.10) and DFS (Fig. 2b, r = 0.26,* p* = 0.06).

GO analysis and GSEA illustrated that the most significantly associated molecular function of the green module was IgG binding (Supplementary Fig. 2a–c), the most enriched biological process was neutrophil activation (Supplementary Fig. 2d–f), and the top-ranking two cellular components were vacuolar membrane and lysosome membrane (Supplementary Fig. 2g). Finally, GSEA showed that the green module was related to endocytosis and soft-tissue sarcomas (Fig. 2c, d).

**Fig. 3:**
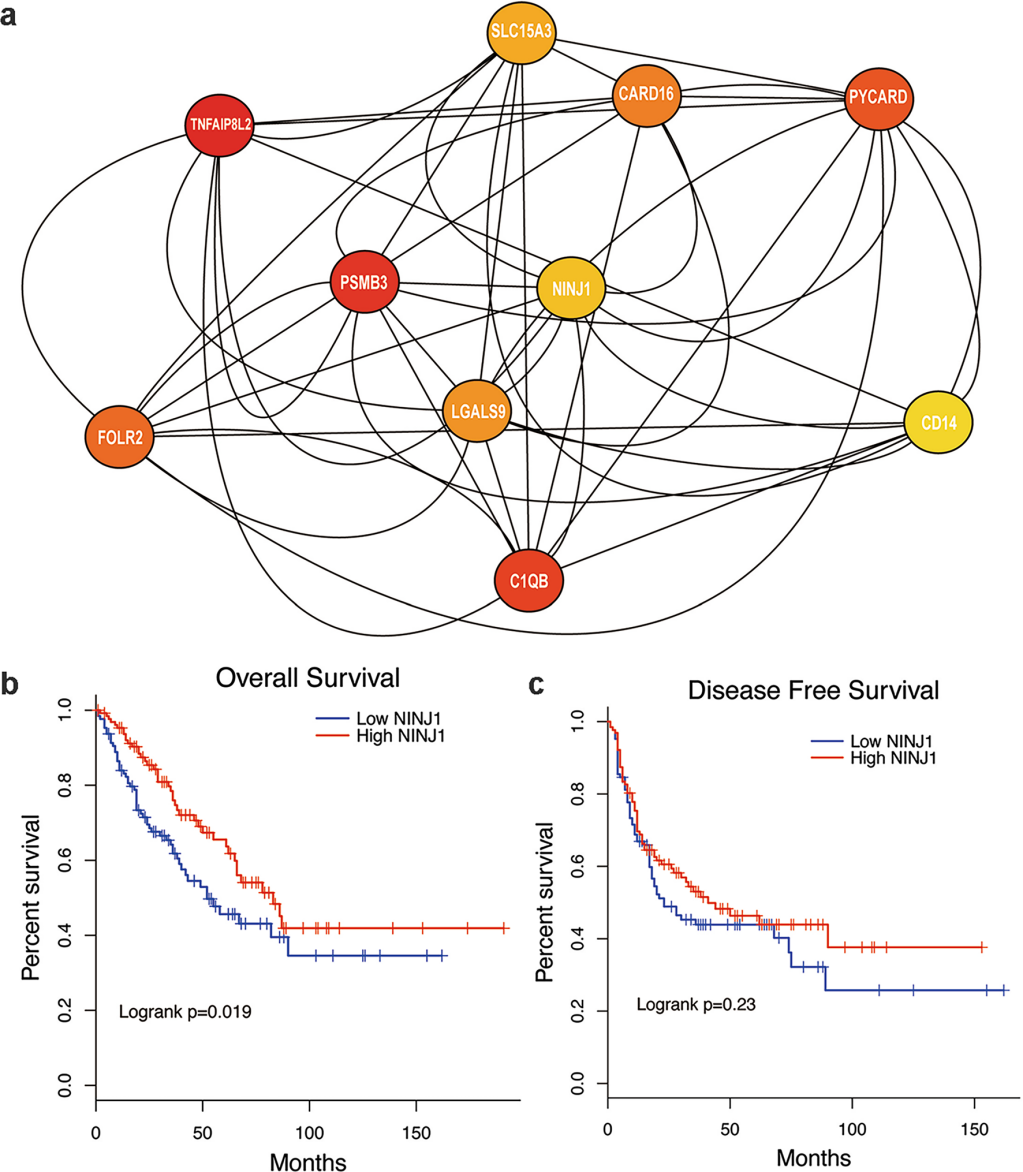
Discovery of prognosis-related hub genes including *NINJ1* in the training cohort and the relationships between *NINJ1* expression levels and disease-free survival (DFS) and overall survival (OS). **a** Hub genes in the green module were selected using the betweenness analysis in Cytoscape software. Patients with higher *NINJ1* expression levels exhibit better OS (**b**) and better DFS (**c**)

We further evaluated the prognostic potential of individual genes in the green module. First, we ranked genes by co-expression correlation. The module rank was established from the relative coefficient between the expression level of a specific gene and the module eigengene, which represented consistency with the overall expression trend of the module. We also calculated the hazard ratio between each gene and OS and DFS (Supplementary Table 1, 2). Hub genes in the green module were subsequently selected using the betweenness analysis of the Cytoscape software. A gene–gene interaction network was constructed to visualize the relationships among these hub genes. The top 10 hub genes in the green module were *NINJ1, SLC15A3, CARD16, PYCARD, CD14, TNFAIP8L2, PSMB3, LGALS9, FOLR2,* and *C1QB* (Fig. 3a). These hub genes exhibited higher correlations with the module eigengene and possessed more interactions with other genes inside the module than did non-hub genes (Betweenness analysis, Supplementary Table 3).

KM analysis showed that, of all the identified hub genes, only higher expression levels of NINJ1 led to the best OS (Fig. 3b, *p* = 0.019) and DFS (Fig. 3c, *p* = 0.23). Thus, NINJ1 was identified as a potential core prognostic predictor because it is not only prognosis-related but is also a hub gene in a prognosis-related gene module.

### External validation

**Fig. 4:**
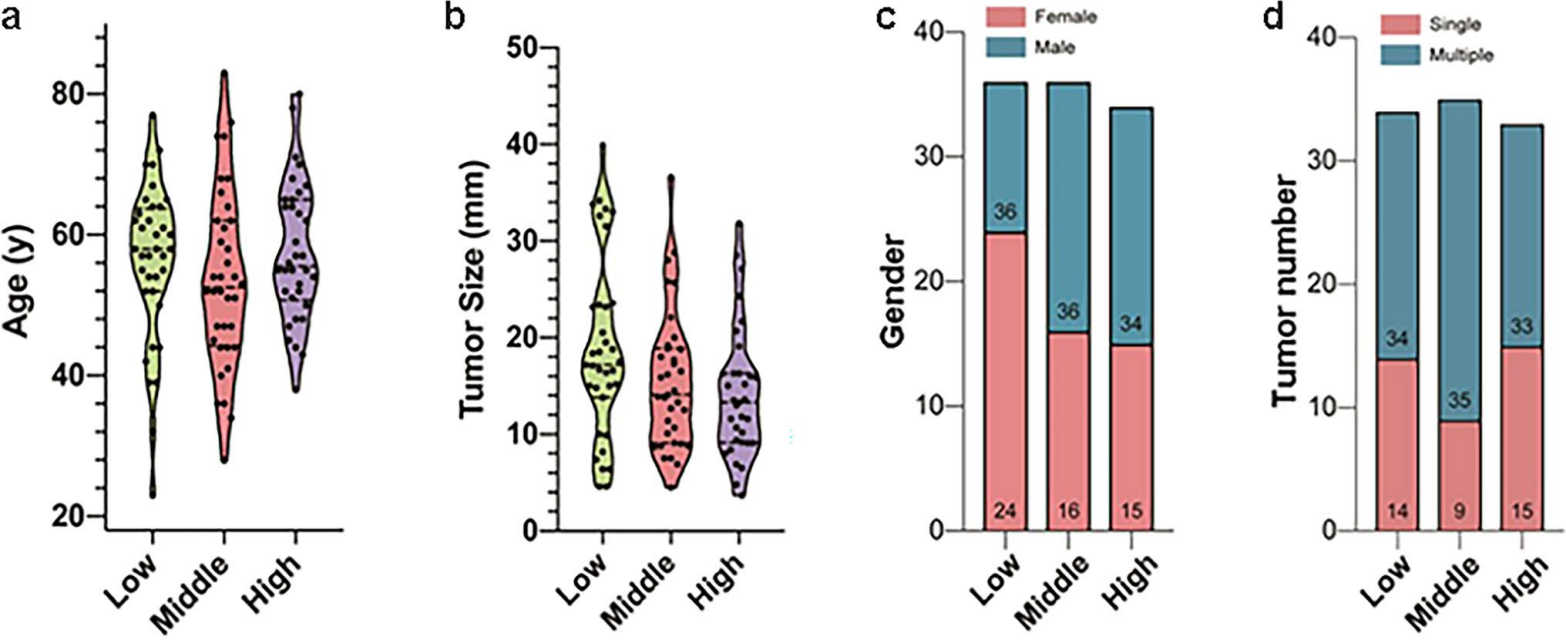
Comparison of different clinical parameters among low, middle, and high NINJ1 expressed RPLS patients

**Fig. 5:**
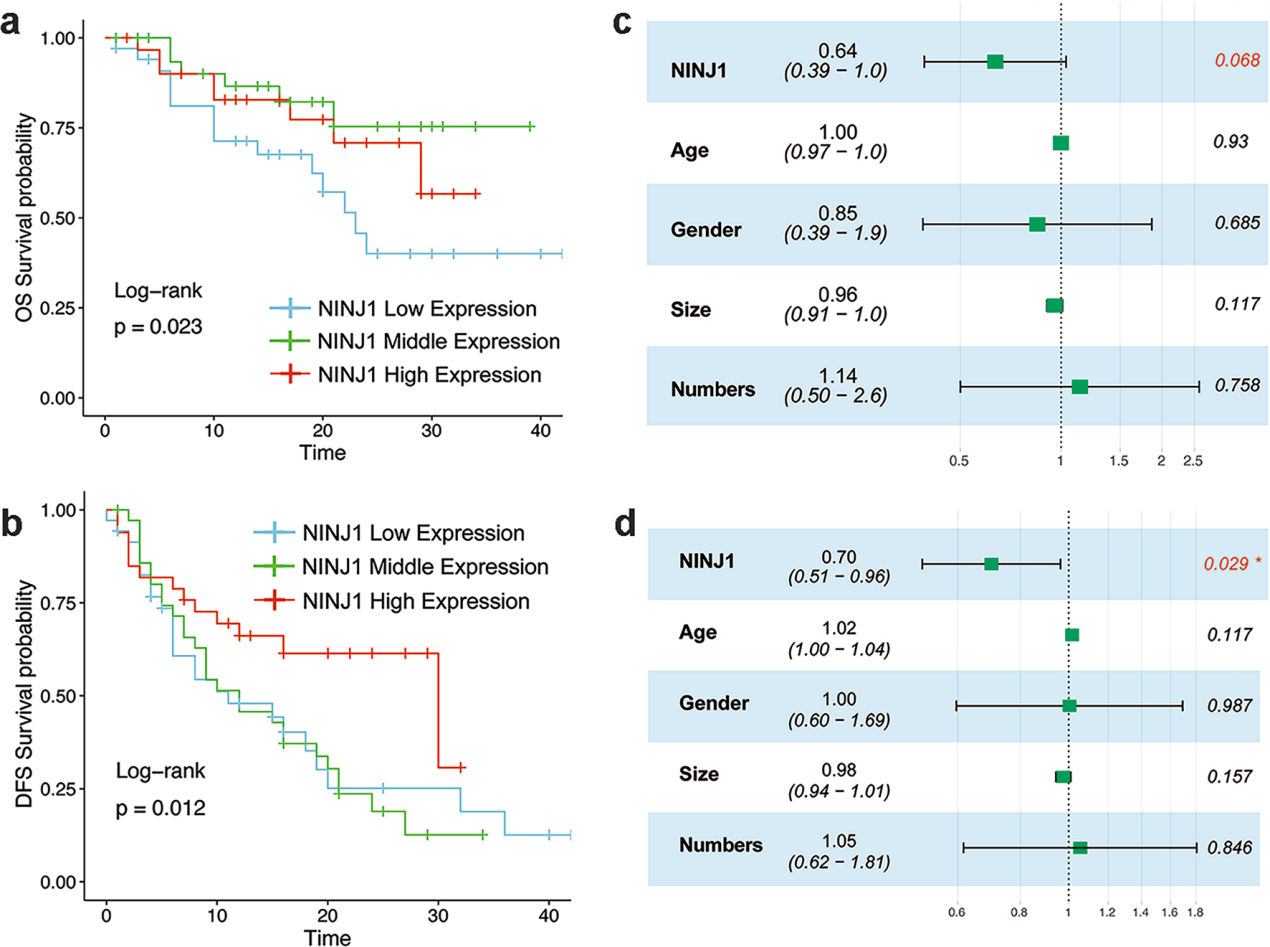
Independent external validation of NINJ1 as a prognostic predictor for RPLS patients. Kaplan–Meier analysis shows a higher survival probability in the NINJ1 high-expressing group in both OS (**a**) and DFS (**b**). Forest plots of Cox models of both OS (**c**) and DFS (**d**). DFS disease-free survival, OS overall survival

Surgery-resected tissues were available for all patients in the external validation group, and the survival data including OS and DFS of each patient were obtained after long-term follow-up (median follow-up time, 42 months). All samples were divided into three groups according to the IHC staining results of NINJ1. No significant differences were observed among these subgroups concerning age, tumor size, gender, and tumor numbers (Fig. 4). When compared with the lower group, the KM analysis suggested a higher survival probability in the NINJ1 high-expressing group in terms of both OS (Fig. 5a, *p* = 0.023) and DFS (Fig. 5b, *p* = 0.012). Multivariable Cox analysis confirmed the independent prognostic predictor status of NINJ1, and it suggested a higher survival probability in both OS (Fig. 5c, *p* = 0.068) and DFS (Fig. 5d, *p* = 0.029). The forest plots of Cox models involving patient age, sex, tumor size, and tumor numbers showed no significant differences among subgroups. Thus, we conclude that NINJ1 is an independent prognostic factor in patients with RPLS.

## Discussion

The literature concerning prognostic biomarkers for RPLS is extremely limited, with only a few have been reported [21]. The main problems in this field have been poor consensus due to the subjectivity of biomarker selection and small sample sizes, which have impeded the development of clinically available biomarkers for RPLS. Here, we addressed these problems by building a TCGA-based classification and screening process for prognostic biomarkers, and by using our large samples to validate the screening results. The widespread recognition of the TCGA dataset and our own large RPLS population with relatively high homogeneity should contribute to the identification of a consensus biomarker for RPLS.

Our results delineated the functional landscape of the genes involved in one module, which partially explained its close correlation with the prognosis of patients with liposarcoma. In this module*, NINJ1, SLC15A3*, and nearly all the hub genes are reportedly involved in inflammation responses/regulation, signal transduction, and angiogenesis [22–24]. It is worth noting that the neutrophil-to-lymphocyte ratio, an indicator of neutrophil activation, is an important unfavorable predictor in sarcoma patients [25]. A meta-analysis has also quantified an association between elevated neutrophil-to-lymphocyte ratio and poor OS (hazard ratio, 1.59; *p* < 0.001) and DFS (hazard ratio, 1.28; *p* < 0.001) in sarcoma patients [26]. Our omics data also supported the opinion that neutrophil activation is a crucial biological process in liposarcoma progression [23]. Furthermore, it has been reported that NINJ1 has two distinct functions in p53-dependent tumorigenesis [27, 28]. NINJ1 represses the translation of both wild-type and mutated *p53* and acts as an oncogene and a tumor-suppressor gene, respectively, in cells with wild-type and mutant *p53*. According to the TCGA database, *p53* is the first-ranked mutated gene among all liposarcoma genes. Thus, it is reasonable that NINJ1 may be a good prognostic factor for liposarcomas.

Our study has some limitations. First, all liposarcomas were included in the gene screening process because of the lack of RPLS-specific samples in the TCGA sarcoma dataset. However, the strict inclusion and exclusion criteria of our validation cohort largely compensate for this deficiency. Second, the size of the TCGA liposarcoma cohort was relatively small considering the large number of potential predictors. Further genomic and transcriptomic datasets from larger numbers of RPLS patients are urgently required. We believe that a larger and more complete RPLS database for biomarker discovery would greatly promote progress in this field.

In conclusion, we established a step-wise screening pipeline to identify credible and specific prognostic biomarkers for RPLS from the TCGA dataset and obtained a series of potential prognostic biomarkers. Notably, NINJ1 emerged as the top-ranked biomarker, significantly associated with both patient prognosis and biological importance. Further verification by IHC with our own RESAR cohort confirmed that NINJ1 was an independent prognostic predictor for RPLS. The identification of NINJ1 as a prognostic factor represents a significant advance in the management of RPLS, with the potential to guide treatment decisions, improve patient outcomes, and inspire the development of novel targeted drugs.

### Supplementary Information

Supplementary Material 1.

Supplementary Material 2.

Supplementary Material 3.

Supplementary Material 4.

## Data Availability

The datasets provided in this study can be downloaded from online repositories. If you have further inquiries, please contact the authors.
