# Supplementary material for: Identification of NINJ1 as a novel prognostic predictor for retroperitoneal liposarcoma
Source: Discov Oncol. 2024 May 11;15:155. doi: 10.1007/s12672-024-01016-x (PMC11088571; doi:10.1007/s12672-024-01016-x)
Supplement: Supplementary file 1 — Supplementary Material 1. [file 12672_2024_1016_MOESM1_ESM.docx]

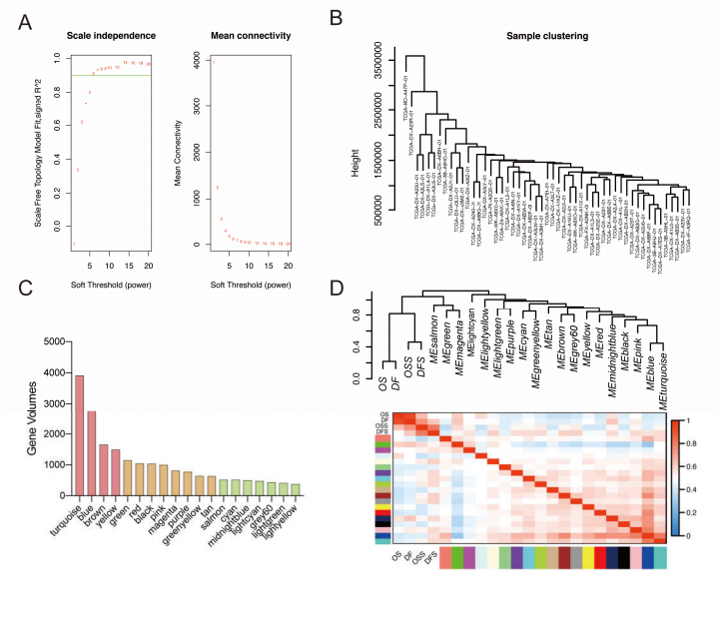


Supplementary Fig. 1 Detailed parameters in WGCNA analysis.

A. Scale independence and soft threshold identification of WGCNA. B. Sample clustering of WGCNA. C. Gene volumes in each gene modules. D. Correlation heatmap of each module and OS/DFS.


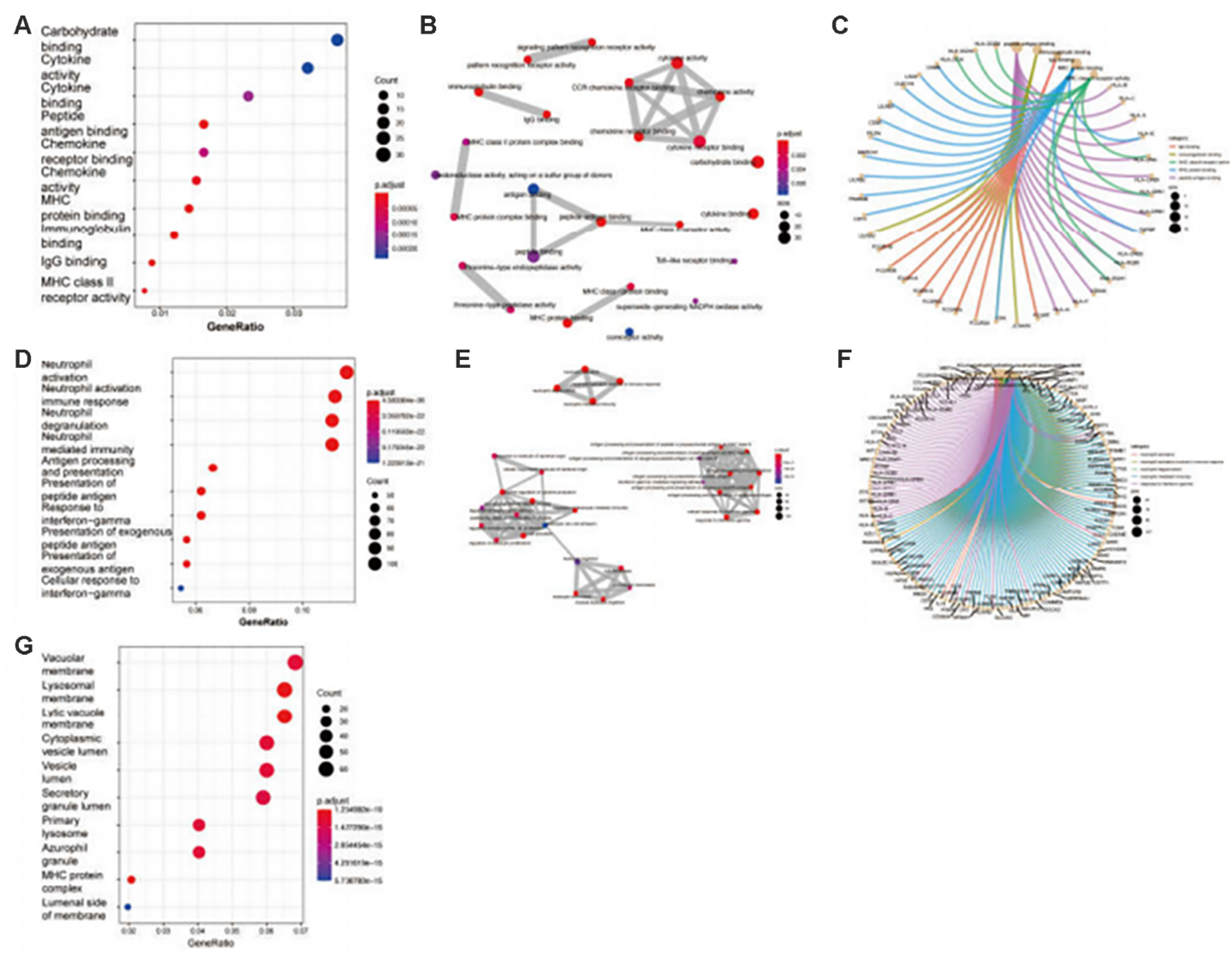


Supplementary Fig. 2 GO analysis and GSEA analysis of green module.

A-C. Molecular function of green module focused on IgG binding. D-F. Biological process of green module is mostly related to neutrophil activation. G. Cellular component of green module is involved in vacuolar membrane and lysosome membrane.
